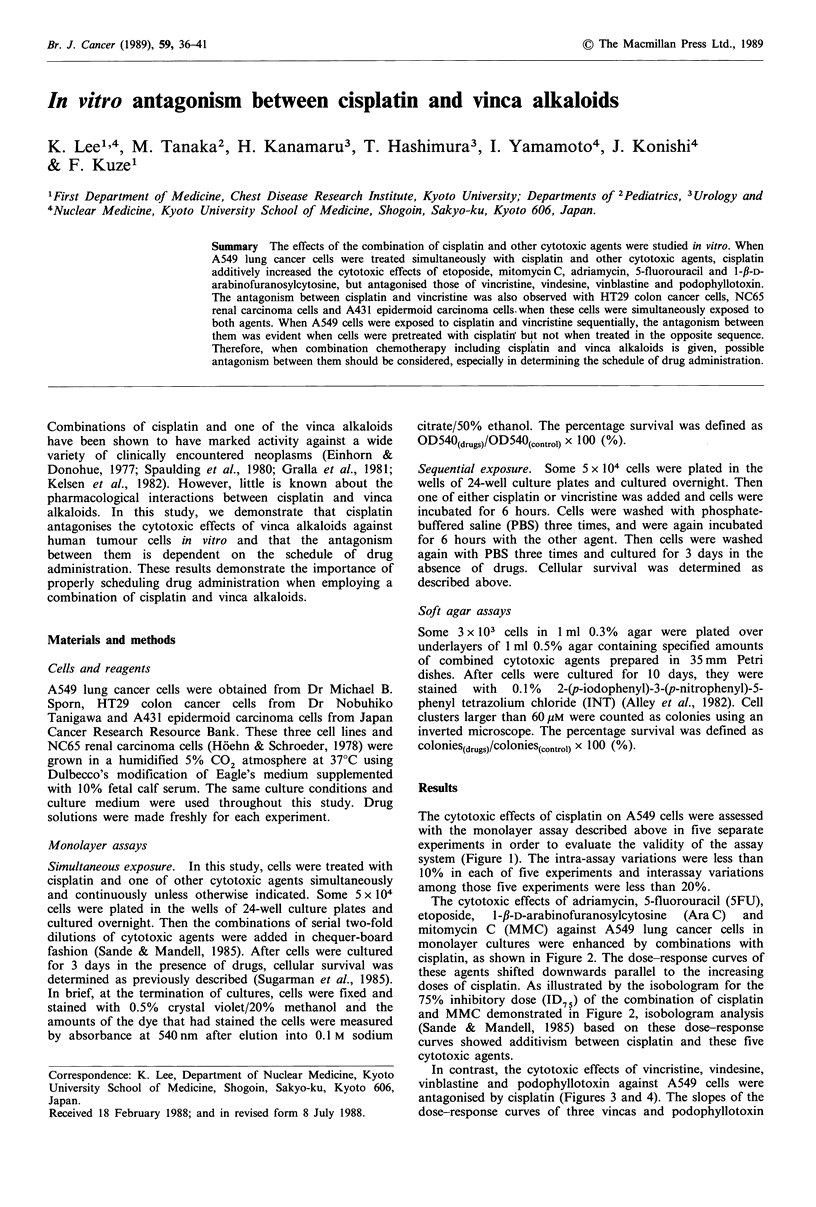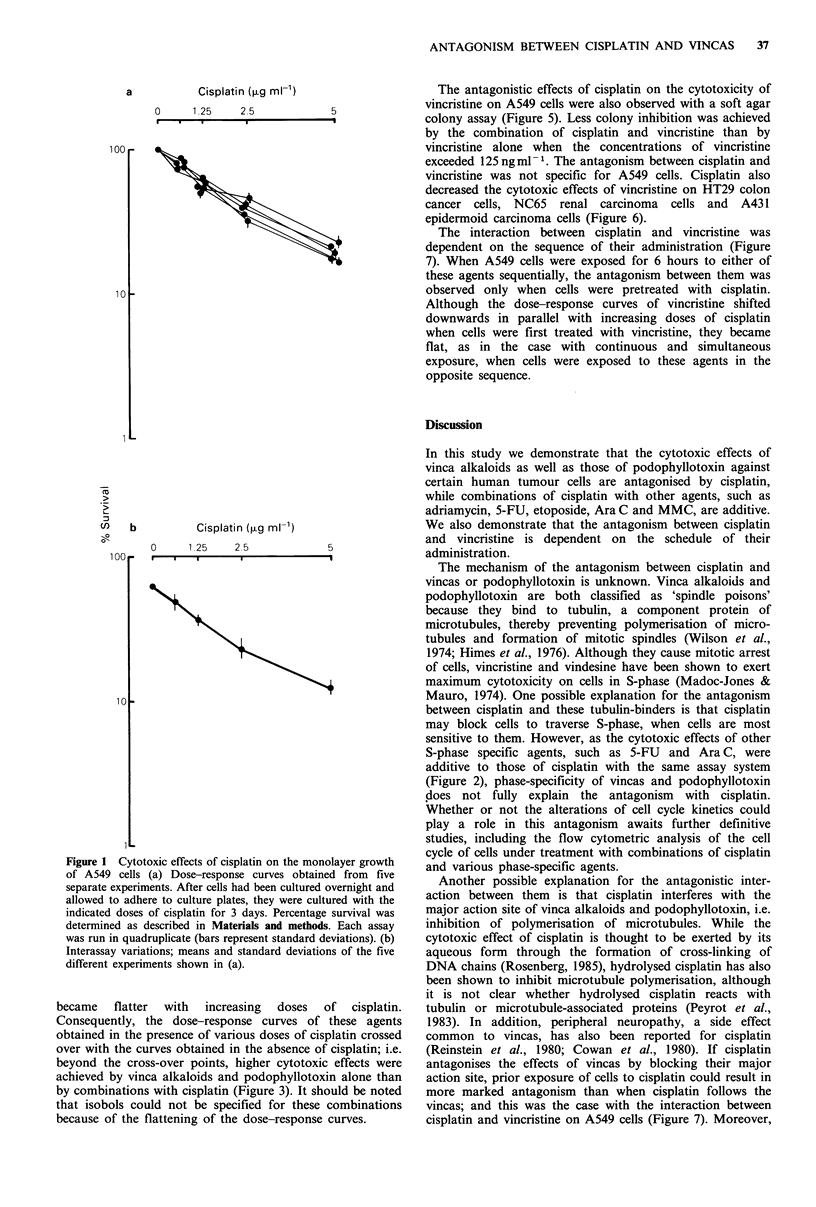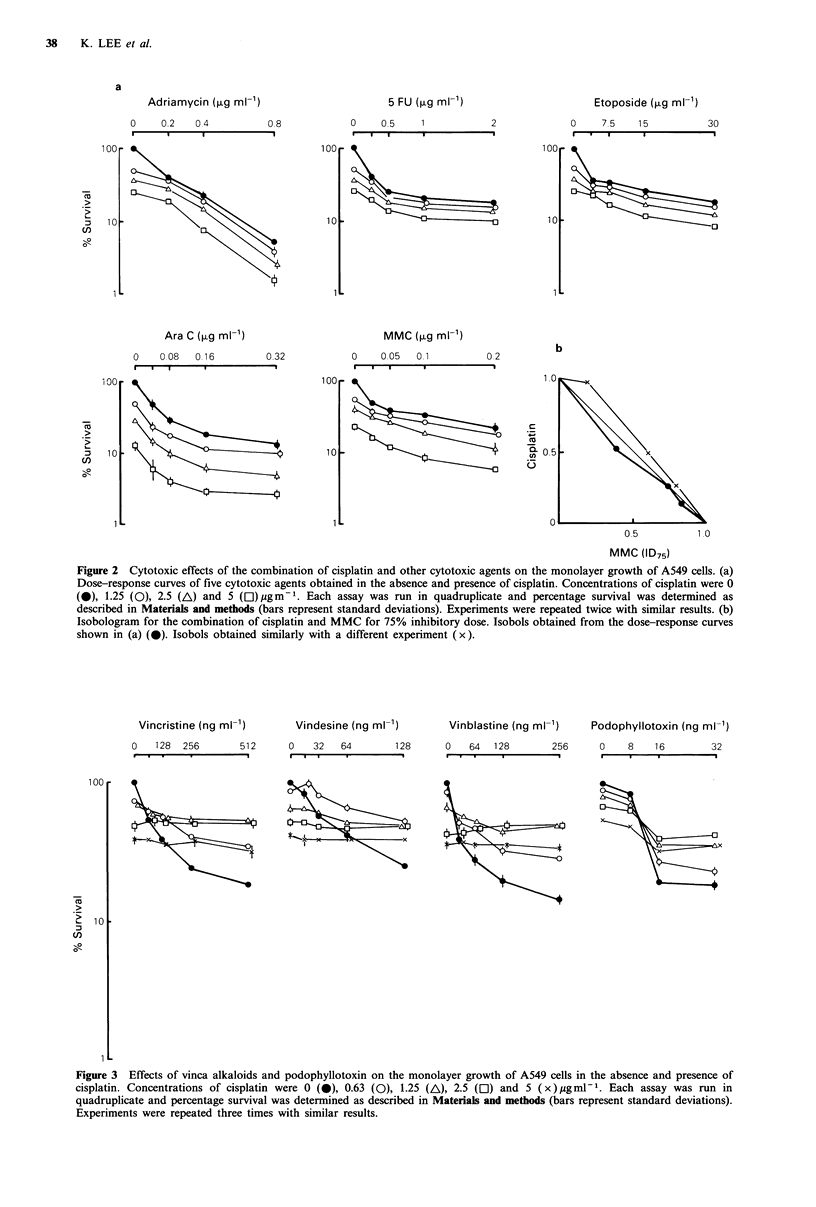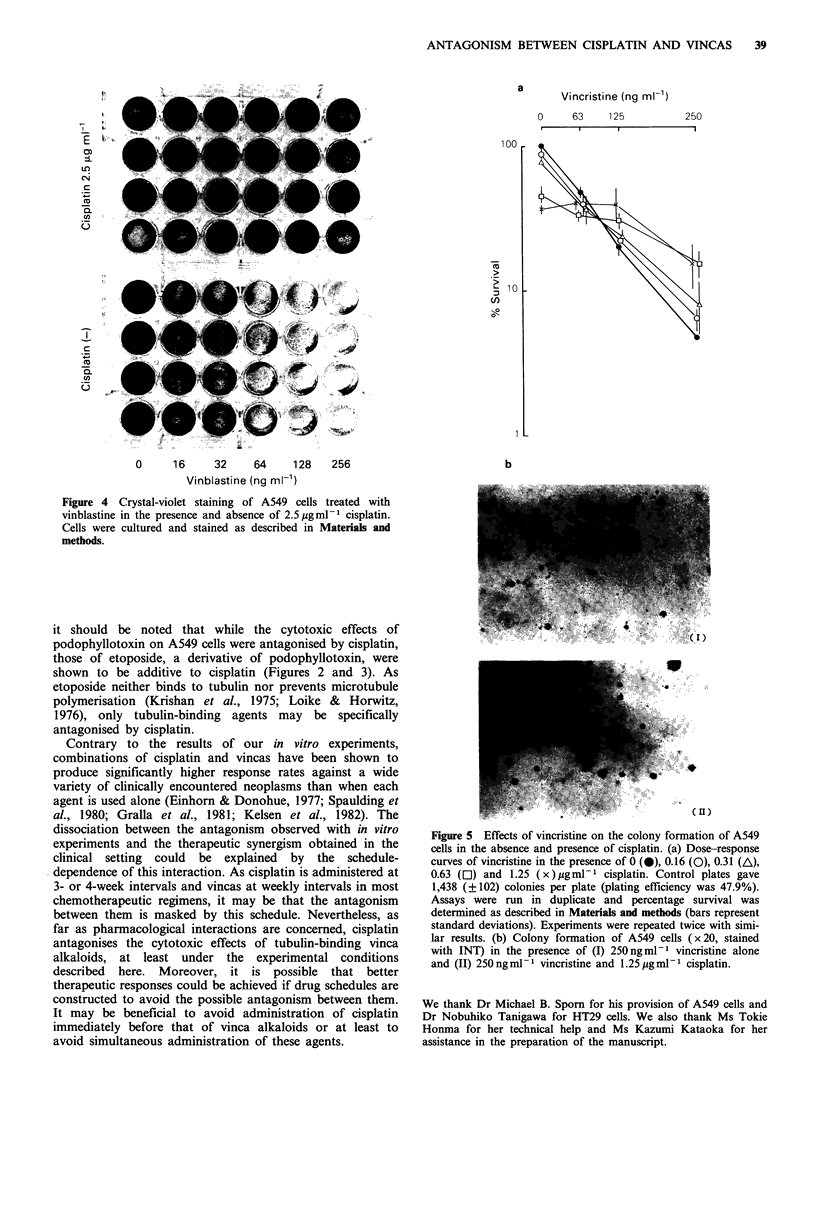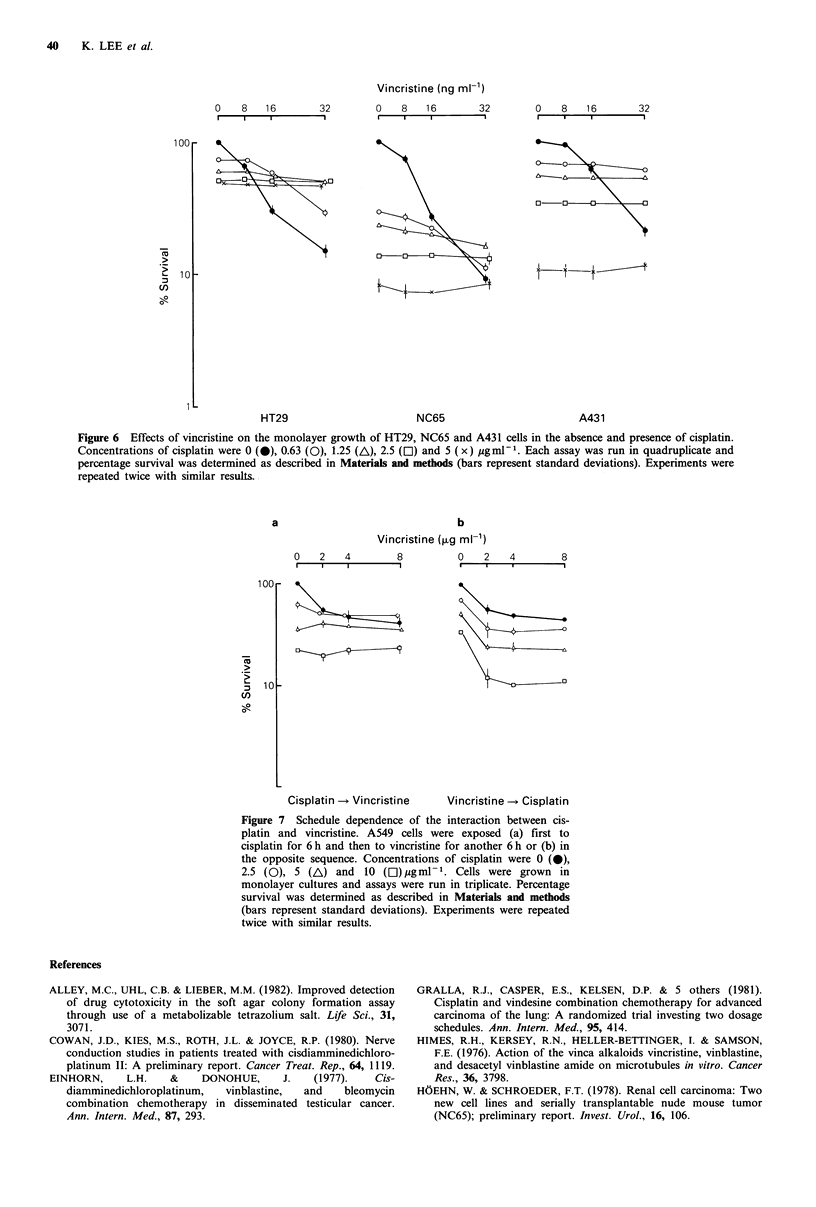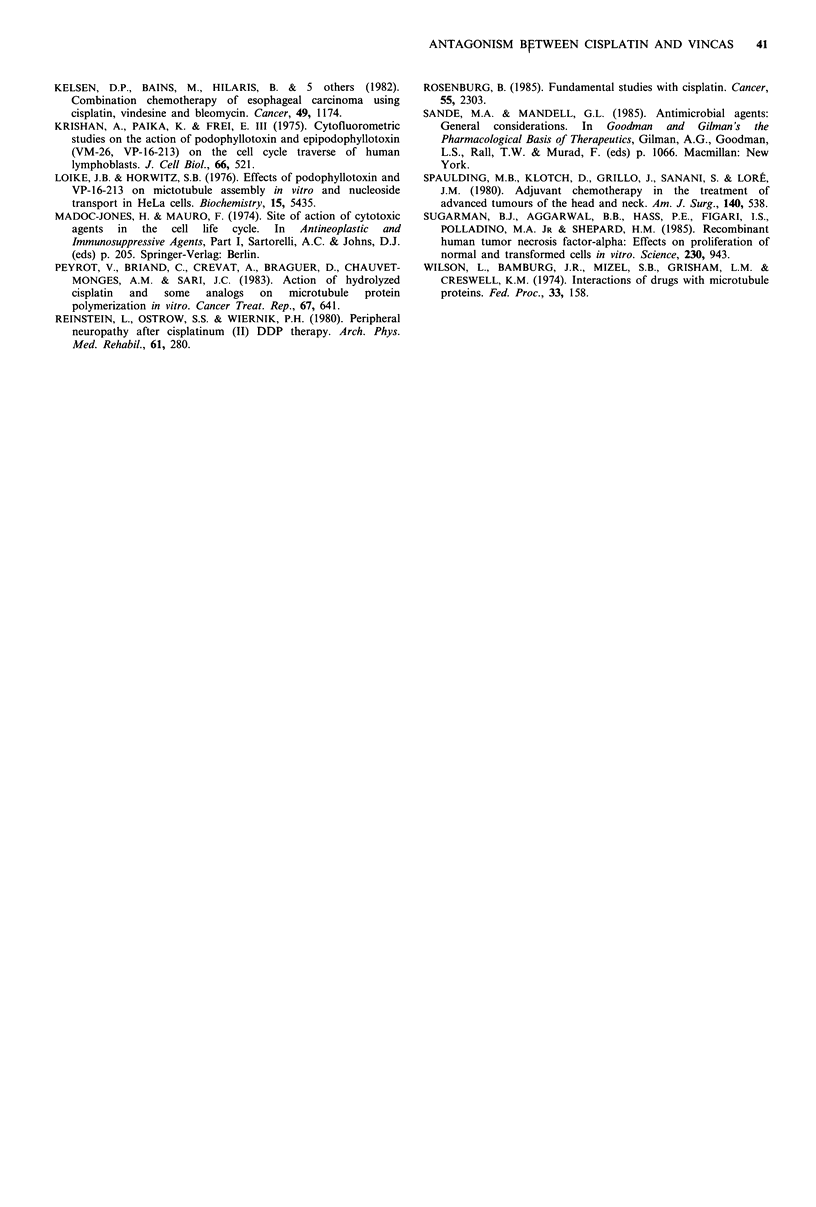# In vitro antagonism between cisplatin and vinca alkaloids.

**DOI:** 10.1038/bjc.1989.8

**Published:** 1989-01

**Authors:** K. Lee, M. Tanaka, H. Kanamaru, T. Hashimura, I. Yamamoto, J. Konishi, F. Kuze

**Affiliations:** First Department of Medicine, Kyoto University, Japan.

## Abstract

**Images:**


Br. J. Cancer (1989), 59, 36–41

© The Macmillan Press Ltd., 1989

### *In vitro* antagonism between cisplatin and vinca alkaloids

K. Lee[1,4], M. Tanaka[2], H. Kanamaru[3], T. Hashimura[3], I. Yamamoto[4], J. Konishi[4] & F. Kuze[1]

[1]*First Department of Medicine, Chest Disease Research Institute, Kyoto University; Departments of* [2]*Pediatrics,* [3]*Urology and* [4]*Nuclear Medicine, Kyoto University School of Medicine, Shogoin, Sakyo-ku, Kyoto 606, Japan.*

**Summary** The effects of the combination of cisplatin and other cytotoxic agents were studied *in vitro*. When A549 lung cancer cells were treated simultaneously with cisplatin and other cytotoxic agents, cisplatin additively increased the cytotoxic effects of etoposide, mitomycin C, adriamycin, 5-fluorouracil and 1-β-D-arabinofuranosylcytosine, but antagonised those of vincristine, vindesine, vinblastine and podophyllotoxin. The antagonism between cisplatin and vincristine was also observed with HT29 colon cancer cells, NC65 renal carcinoma cells and A431 epidermoid carcinoma cells when these cells were simultaneously exposed to both agents. When A549 cells were exposed to cisplatin and vincristine sequentially, the antagonism between them was evident when cells were pretreated with cisplatin but not when treated in the opposite sequence. Therefore, when combination chemotherapy including cisplatin and vinca alkaloids is given, possible antagonism between them should be considered, especially in determining the schedule of drug administration.

Combinations of cisplatin and one of the vinca alkaloids have been shown to have marked activity against a wide variety of clinically encountered neoplasms (Einhorn & Donohue, 1977; Spaulding *et al.*, 1980; Gralla *et al.*, 1981; Kelsen *et al.*, 1982). However, little is known about the pharmacological interactions between cisplatin and vinca alkaloids. In this study, we demonstrate that cisplatin antagonises the cytotoxic effects of vinca alkaloids against human tumour cells *in vitro* and that the antagonism between them is dependent on the schedule of drug administration. These results demonstrate the importance of properly scheduling drug administration when employing a combination of cisplatin and vinca alkaloids.

### Materials and methods

### *Cells and reagents*

A549 lung cancer cells were obtained from Dr Michael B. Sporn, HT29 colon cancer cells from Dr Nobuhiko Tanigawa and A431 epidermoid carcinoma cells from Japan Cancer Research Resource Bank. These three cell lines and NC65 renal carcinoma cells (Höehn & Schroeder, 1978) were grown in a humidified 5% $CO_2$ atmosphere at 37°C using Dulbecco's modification of Eagle's medium supplemented with 10% fetal calf serum. The same culture conditions and culture medium were used throughout this study. Drug solutions were made freshly for each experiment.

### *Monolayer assays*

*Simultaneous exposure.* In this study, cells were treated with cisplatin and one of other cytotoxic agents simultaneously and continuously unless otherwise indicated. Some $5 \times 10^4$ cells were plated in the wells of 24-well culture plates and cultured overnight. Then the combinations of serial two-fold dilutions of cytotoxic agents were added in chequer-board fashion (Sande & Mandell, 1985). After cells were cultured for 3 days in the presence of drugs, cellular survival was determined as previously described (Sugarman *et al.*, 1985). In brief, at the termination of cultures, cells were fixed and stained with 0.5% crystal violet/20% methanol and the amounts of the dye that had stained the cells were measured by absorbance at 540 nm after elution into 0.1 M sodium citrate/50% ethanol. The percentage survival was defined as $OD540_{(drugs)}/OD540_{(control)} \times 100$ (%).

*Sequential exposure.* Some $5 \times 10^4$ cells were plated in the wells of 24-well culture plates and cultured overnight. Then one of either cisplatin or vincristine was added and cells were incubated for 6 hours. Cells were washed with phosphate-buffered saline (PBS) three times, and were again incubated for 6 hours with the other agent. Then cells were washed again with PBS three times and cultured for 3 days in the absence of drugs. Cellular survival was determined as described above.

### *Soft agar assays*

Some $3 \times 10^3$ cells in 1 ml 0.3% agar were plated over underlayers of 1 ml 0.5% agar containing specified amounts of combined cytotoxic agents prepared in 35 mm Petri dishes. After cells were cultured for 10 days, they were stained with 0.1% 2-(*p*-iodophenyl)-3-(*p*-nitrophenyl)-5-phenyl tetrazolium chloride (INT) (Alley *et al.*, 1982). Cell clusters larger than 60 μM were counted as colonies using an inverted microscope. The percentage survival was defined as $colonies_{(drugs)}/colonies_{(control)} \times 100$ (%).

### Results

The cytotoxic effects of cisplatin on A549 cells were assessed with the monolayer assay described above in five separate experiments in order to evaluate the validity of the assay system (Figure 1). The intra-assay variations were less than 10% in each of five experiments and interassay variations among those five experiments were less than 20%.

The cytotoxic effects of adriamycin, 5-fluorouracil (5FU), etoposide, 1-β-D-arabinofuranosylcytosine (Ara C) and mitomycin C (MMC) against A549 lung cancer cells in monolayer cultures were enhanced by combinations with cisplatin, as shown in Figure 2. The dose–response curves of these agents shifted downwards parallel to the increasing doses of cisplatin. As illustrated by the isobologram for the 75% inhibitory dose ($ID_{75}$) of the combination of cisplatin and MMC demonstrated in Figure 2, isobologram analysis (Sande & Mandell, 1985) based on these dose–response curves showed additivism between cisplatin and these five cytotoxic agents.

In contrast, the cytotoxic effects of vincristine, vindesine, vinblastine and podophyllotoxin against A549 cells were antagonised by cisplatin (Figures 3 and 4). The slopes of the dose–response curves of three vincas and podophyllotoxin

Correspondence: K. Lee, Department of Nuclear Medicine, Kyoto University School of Medicine, Shogoin, Sakyo-ku, Kyoto 606, Japan.

Received 18 February 1988; and in revised form 8 July 1988.

**Figure 1** Cytotoxic effects of cisplatin on the monolayer growth of A549 cells (a) Dose–response curves obtained from five separate experiments. After cells had been cultured overnight and allowed to adhere to culture plates, they were cultured with the indicated doses of cisplatin for 3 days. Percentage survival was determined as described in **Materials and methods**. Each assay was run in quadruplicate (bars represent standard deviations). (b) Interassay variations; means and standard deviations of the five different experiments shown in (a).

became flatter with increasing doses of cisplatin. Consequently, the dose–response curves of these agents obtained in the presence of various doses of cisplatin crossed over with the curves obtained in the absence of cisplatin; i.e. beyond the cross-over points, higher cytotoxic effects were achieved by vinca alkaloids and podophyllotoxin alone than by combinations with cisplatin (Figure 3). It should be noted that isobols could not be specified for these combinations because of the flattening of the dose–response curves.

The antagonistic effects of cisplatin on the cytotoxicity of vincristine on A549 cells were also observed with a soft agar colony assay (Figure 5). Less colony inhibition was achieved by the combination of cisplatin and vincristine than by vincristine alone when the concentrations of vincristine exceeded 125 ng ml$^{-1}$. The antagonism between cisplatin and vincristine was not specific for A549 cells. Cisplatin also decreased the cytotoxic effects of vincristine on HT29 colon cancer cells, NC65 renal carcinoma cells and A431 epidermoid carcinoma cells (Figure 6).

The interaction between cisplatin and vincristine was dependent on the sequence of their administration (Figure 7). When A549 cells were exposed for 6 hours to either of these agents sequentially, the antagonism between them was observed only when cells were pretreated with cisplatin. Although the dose–response curves of vincristine shifted downwards in parallel with increasing doses of cisplatin when cells were first treated with vincristine, they became flat, as in the case with continuous and simultaneous exposure, when cells were exposed to these agents in the opposite sequence.

### Discussion

In this study we demonstrate that the cytotoxic effects of vinca alkaloids as well as those of podophyllotoxin against certain human tumour cells are antagonised by cisplatin, while combinations of cisplatin with other agents, such as adriamycin, 5-FU, etoposide, Ara C and MMC, are additive. We also demonstrate that the antagonism between cisplatin and vincristine is dependent on the schedule of their administration.

The mechanism of the antagonism between cisplatin and vincas or podophyllotoxin is unknown. Vinca alkaloids and podophyllotoxin are both classified as 'spindle poisons' because they bind to tubulin, a component protein of microtubules, thereby preventing polymerisation of microtubules and formation of mitotic spindles (Wilson *et al.*, 1974; Himes *et al.*, 1976). Although they cause mitotic arrest of cells, vincristine and vindesine have been shown to exert maximum cytotoxicity on cells in S-phase (Madoc-Jones & Mauro, 1974). One possible explanation for the antagonism between cisplatin and these tubulin-binders is that cisplatin may block cells to traverse S-phase, when cells are most sensitive to them. However, as the cytotoxic effects of other S-phase specific agents, such as 5-FU and Ara C, were additive to those of cisplatin with the same assay system (Figure 2), phase-specificity of vincas and podophyllotoxin does not fully explain the antagonism with cisplatin. Whether or not the alterations of cell cycle kinetics could play a role in this antagonism awaits further definitive studies, including the flow cytometric analysis of the cell cycle of cells under treatment with combinations of cisplatin and various phase-specific agents.

Another possible explanation for the antagonistic interaction between them is that cisplatin interferes with the major action site of vinca alkaloids and podophyllotoxin, i.e. inhibition of polymerisation of microtubules. While the cytotoxic effect of cisplatin is thought to be exerted by its aqueous form through the formation of cross-linking of DNA chains (Rosenberg, 1985), hydrolysed cisplatin has also been shown to inhibit microtubule polymerisation, although it is not clear whether hydrolysed cisplatin reacts with tubulin or microtubule-associated proteins (Peyrot *et al.*, 1983). In addition, peripheral neuropathy, a side effect common to vincas, has also been reported for cisplatin (Reinstein *et al.*, 1980; Cowan *et al.*, 1980). If cisplatin antagonises the effects of vincas by blocking their major action site, prior exposure of cells to cisplatin could result in more marked antagonism than when cisplatin follows the vincas; and this was the case with the interaction between cisplatin and vincristine on A549 cells (Figure 7). Moreover,

**Figure 2** Cytotoxic effects of the combination of cisplatin and other cytotoxic agents on the monolayer growth of A549 cells. (a) Dose–response curves of five cytotoxic agents obtained in the absence and presence of cisplatin. Concentrations of cisplatin were 0 (●), 1.25 (○), 2.5 (△) and 5 (□) $\mu g m^{-1}$. Each assay was run in quadruplicate and percentage survival was determined as described in **Materials and methods** (bars represent standard deviations). Experiments were repeated twice with similar results. (b) Isobologram for the combination of cisplatin and MMC for 75% inhibitory dose. Isobols obtained from the dose–response curves shown in (a) (●). Isobols obtained similarly with a different experiment (×).

**Figure 3** Effects of vinca alkaloids and podophyllotoxin on the monolayer growth of A549 cells in the absence and presence of cisplatin. Concentrations of cisplatin were 0 (●), 0.63 (○), 1.25 (△), 2.5 (□) and 5 (×) $\mu g ml^{-1}$. Each assay was run in quadruplicate and percentage survival was determined as described in **Materials and methods** (bars represent standard deviations). Experiments were repeated three times with similar results.

**Figure 4** Crystal-violet staining of A549 cells treated with vinblastine in the presence and absence of 2.5 $\mu g ml^{-1}$ cisplatin. Cells were cultured and stained as described in **Materials and methods**.

it should be noted that while the cytotoxic effects of podophyllotoxin on A549 cells were antagonised by cisplatin, those of etoposide, a derivative of podophyllotoxin, were shown to be additive to cisplatin (Figures 2 and 3). As etoposide neither binds to tubulin nor prevents microtubule polymerisation (Krishan *et al.*, 1975; Loike & Horwitz, 1976), only tubulin-binding agents may be specifically antagonised by cisplatin.

Contrary to the results of our *in vitro* experiments, combinations of cisplatin and vincas have been shown to produce significantly higher response rates against a wide variety of clinically encountered neoplasms than when each agent is used alone (Einhorn & Donohue, 1977; Spaulding *et al.*, 1980; Gralla *et al.*, 1981; Kelsen *et al.*, 1982). The dissociation between the antagonism observed with *in vitro* experiments and the therapeutic synergism obtained in the clinical setting could be explained by the schedule-dependence of this interaction. As cisplatin is administered at 3- or 4-week intervals and vincas at weekly intervals in most chemotherapeutic regimens, it may be that the antagonism between them is masked by this schedule. Nevertheless, as far as pharmacological interactions are concerned, cisplatin antagonises the cytotoxic effects of tubulin-binding vinca alkaloids, at least under the experimental conditions described here. Moreover, it is possible that better therapeutic responses could be achieved if drug schedules are constructed to avoid the possible antagonism between them. It may be beneficial to avoid administration of cisplatin immediately before that of vinca alkaloids or at least to avoid simultaneous administration of these agents.

**Figure 5** Effects of vincristine on the colony formation of A549 cells in the absence and presence of cisplatin. (a) Dose–response curves of vincristine in the presence of 0 (●), 0.16 (○), 0.31 (△), 0.63 (□) and 1.25 (×) $\mu g ml^{-1}$ cisplatin. Control plates gave 1,438 (±102) colonies per plate (plating efficiency was 47.9%). Assays were run in duplicate and percentage survival was determined as described in **Materials and methods** (bars represent standard deviations). Experiments were repeated twice with similar results. (b) Colony formation of A549 cells (×20, stained with INT) in the presence of (I) 250 $ng ml^{-1}$ vincristine alone and (II) 250 $ng ml^{-1}$ vincristine and 1.25 $\mu g ml^{-1}$ cisplatin.

We thank Dr Michael B. Sporn for his provision of A549 cells and Dr Nobuhiko Tanigawa for HT29 cells. We also thank Ms Tokie Honma for her technical help and Ms Kazumi Kataoka for her assistance in the preparation of the manuscript.

**Figure 6** Effects of vincristine on the monolayer growth of HT29, NC65 and A431 cells in the absence and presence of cisplatin. Concentrations of cisplatin were 0 (●), 0.63 (○), 1.25 (△), 2.5 (□) and 5 (×) $\mu$g ml$^{-1}$. Each assay was run in quadruplicate and percentage survival was determined as described in **Materials and methods** (bars represent standard deviations). Experiments were repeated twice with similar results.

**Figure 7** Schedule dependence of the interaction between cisplatin and vincristine. A549 cells were exposed (a) first to cisplatin for 6 h and then to vincristine for another 6 h or (b) in the opposite sequence. Concentrations of cisplatin were 0 (●), 2.5 (○), 5 (△) and 10 (□) $\mu$g ml$^{-1}$. Cells were grown in monolayer cultures and assays were run in triplicate. Percentage survival was determined as described in **Materials and methods** (bars represent standard deviations). Experiments were repeated twice with similar results.

### References

ALLEY, M.C., UHL, C.B. & LIEBER, M.M. (1982). Improved detection of drug cytotoxicity in the soft agar colony formation assay through use of a metabolizable tetrazolium salt. *Life Sci.*, **31**, 3071.

COWAN, J.D., KIES, M.S., ROTH, J.L. & JOYCE, R.P. (1980). Nerve conduction studies in patients treated with cisdiamminedichloroplatinum II: A preliminary report. *Cancer Treat. Rep.*, **64**, 1119.

EINHORN, L.H. & DONOHUE, J. (1977). *Cis*-diamminedichloroplatinum, vinblastine, and bleomycin combination chemotherapy in disseminated testicular cancer. *Ann. Intern. Med.*, **87**, 293.

GRALLA, R.J., CASPER, E.S., KELSEN, D.P. & 5 others (1981). Cisplatin and vindesine combination chemotherapy for advanced carcinoma of the lung: A randomized trial investing two dosage schedules. *Ann. Intern. Med.*, **95**, 414.

HIMES, R.H., KERSEY, R.N., HELLER-BETTINGER, I. & SAMSON, F.E. (1976). Action of the vinca alkaloids vincristine, vinblastine, and desacetyl vinblastine amide on microtubules *in vitro*. *Cancer Res.*, **36**, 3798.

HÖEHN, W. & SCHROEDER, F.T. (1978). Renal cell carcinoma: Two new cell lines and serially transplantable nude mouse tumor (NC65); preliminary report. *Invest. Urol.*, **16**, 106.

KELSEN, D.P., BAINS, M., HILARIS, B. & 5 others (1982). Combination chemotherapy of esophageal carcinoma using cisplatin, vindesine and bleomycin. *Cancer*, **49**, 1174.

KRISHAN, A., PAIKA, K. & FREI, E. III (1975). Cytofluorometric studies on the action of podophyllotoxin and epipodophyllotoxin (VM-26, VP-16-213) on the cell cycle traverse of human lymphoblasts. *J. Cell Biol.*, **66**, 521.

LOIKE, J.B. & HORWITZ, S.B. (1976). Effects of podophyllotoxin and VP-16-213 on mictotubule assembly *in vitro* and nucleoside transport in HeLa cells. *Biochemistry*, **15**, 5435.

MADOC-JONES, H. & MAURO, F. (1974). Site of action of cytotoxic agents in the cell life cycle. In *Antineoplastic and Immunosuppressive Agents*, Part I, Sartorelli, A.C. & Johns, D.J. (eds) p. 205. Springer-Verlag: Berlin.

PEYROT, V., BRIAND, C., CREVAT, A., BRAGUER, D., CHAUVET-MONGES, A.M. & SARI, J.C. (1983). Action of hydrolyzed cisplatin and some analogs on microtubule protein polymerization *in vitro*. *Cancer Treat. Rep.*, **67**, 641.

REINSTEIN, L., OSTROW, S.S. & WIERNIK, P.H. (1980). Peripheral neuropathy after cisplatinum (II) DDP therapy. *Arch. Phys. Med. Rehabil.*, **61**, 280.

ROSENBURG, B. (1985). Fundamental studies with cisplatin. *Cancer*, **55**, 2303.

SANDE, M.A. & MANDELL, G.L. (1985). Antimicrobial agents: General considerations. In *Goodman and Gilman's the Pharmacological Basis of Therapeutics*, Gilman, A.G., Goodman, L.S., Rall, T.W. & Murad, F. (eds) p. 1066. Macmillan: New York.

SPAULDING, M.B., KLOTCH, D., GRILLO, J., SANANI, S. & LORÉ, J.M. (1980). Adjuvant chemotherapy in the treatment of advanced tumours of the head and neck. *Am. J. Surg.*, **140**, 538.

SUGARMAN, B.J., AGGARWAL, B.B., HASS, P.E., FIGARI, I.S., POLLADINO, M.A. JR & SHEPARD, H.M. (1985). Recombinant human tumor necrosis factor-alpha: Effects on proliferation of normal and transformed cells *in vitro*. *Science*, **230**, 943.

WILSON, L., BAMBURG, J.R., MIZEL, S.B., GRISHAM, L.M. & CRESWELL, K.M. (1974). Interactions of drugs with microtubule proteins. *Fed. Proc.*, **33**, 158.